# *Tinea faciei* on the right eyebrow caused by Trichophyton interdigitale*

**DOI:** 10.1590/abd1806-4841.20165270

**Published:** 2016

**Authors:** Kai Wen Zhuang, Ya Ling Dai, Yu Ping Ran, Jebina Lama, Yi Ming Fan

**Affiliations:** 1 West China Hospital, Sichuan University – Sichuan, China; 2 Affiliated Hospital of Guangdong Medical University – Zhanjiang, China

**Keywords:** Eyebrow, Tinea faciei, *Trichophyton mentagrophytes* complex, *Trichophyton interdigitale*, Terbinafine

## Abstract

Tinea faciei is a relatively uncommon dermatophyte infection entailing atypical clinical symptoms, usually misdiagnosed and treated with corticosteroids. The authors describe a case of tinea faciei on the right eyebrow caused by *Trichophyton interdigitale*. The patient was an 18-year-old girl, who had an inflammatory plaque with a scaly, pustular surface on the right eyebrow and upper eyelid, which had persisted for over 1 month. She was once misdiagnosed as having eczema and was treated using corticosteroid cream. A diagnosis of tinea faciei was made based on direct microscopy and culture. The sequencing of the nuclear ribosomal ITS region and β-tubulin gene of the isolate established its *T. interdigitale* lineage. The patient was cured by treatment with systemic terbinafine in combination with topical application of 1% naftifine-0.25% ketaconazole cream for 2 weeks.

## INTRODUCTION

Tinea faciei is a relatively rare dermatophyte infection that occurs on the non-bearded regions of the face. The condition is usually misdiagnosed due to its atypical clinical symptoms. Treatment with corticosteroids makes its presentations incognito.^1^ The most frequent agents of the infection are *Trichophyton mentagrophytes* complex, followed by *Microsporum canis* and *Microsporum gypseum*.^2^ *T. mentagrophytes* complex consists of several anamorphs and three teleomorphs (*Arthroderma vanbreuseghemii, A. benhamiae*, and *A. simii*) and are usually isolated from pets, such as guinea pigs and rabbits.^3^ This fungus can cause inflammatory tinea corporis, tinea faciei and tinea capitis in humans. Here, we report a case of tinea faciei due to *Trichophyton interdigitale*, an anamorph of *A. vanbreuseghemii*.

## CASE REPORT

**Figure 1:**
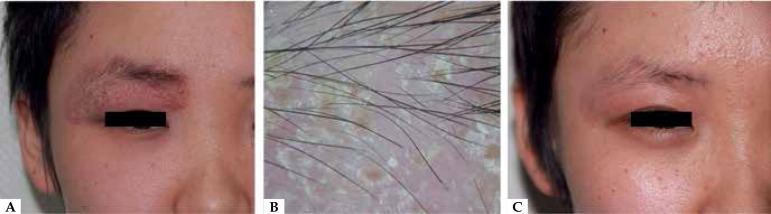
**A**. A deep, intense, inflammatory plaque with a scaly, pustular surface on the 18-year-old girl's right eyebrow and upper eyelid. **B**. The dermoscopy revealed considerable scaling and numerous tiny yellow crusts attached to the lesion's surface and a slight loss of eyebrows. **C**. The inflammatory plaque disappeared after 1 month of antifungal and anti-inflammatory treatment.

An 18-year-old girl presented with a 5-week history of a facial eruption on her right eyebrow and upper eyelid. The lesion began as tiny pustules on a pruritic, erythematous background and was initially diagnosed as eczema. Two ointments containing desonide and mometasone furoate were respectively prescribed. This treatment resulted in fading of the skin lesion, without preventing its progression. After treatment discontinuation, a deep, intense, inflammatory plaque with a scaly, pustular surface recurred (Figure 1A). A slight loss of eyebrows was noted and the dermoscopy revealed considerable scaling and numerous tiny yellow crusts attached to the lesion's surface, a slight loss of eyebrows, while the hair shafts were intact (Figure 1B). Following a detailed inquiry, it emerged that the patient was a college student living with her room-mates in a dormitory, where a pet rabbit had been kept for 2 months. Before her eruption, one of her roommates had developed a similar inflammatory plaque on the left breast, cured by antifungal cream. However, it could not be confirmed that the rabbit was fungal in origin since it died before the patient was referred to our department.

**Figure 2:**
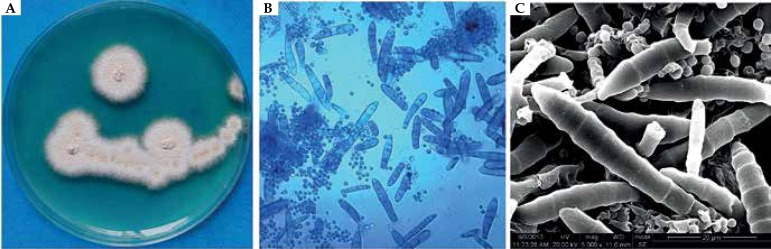
**A**. The colony isolated from the 18-year-old girl on Sabouraud dextrose agar at 28°C for 7 days, showing a beige, powdery surface with orange-yellow pigment. **B**. Microscopic findings revealed a large number of spherial microconidia and thin-walled, clavate and laevis macroconidia without spiral hyphae (lactophenol cotton-blue staining, X 400). **C**. The scanning electron microscopy revealed numerous spherial microconidia and clavate macroconidia with laevis cell walls and protruding ring structures.

**Figure 3:**
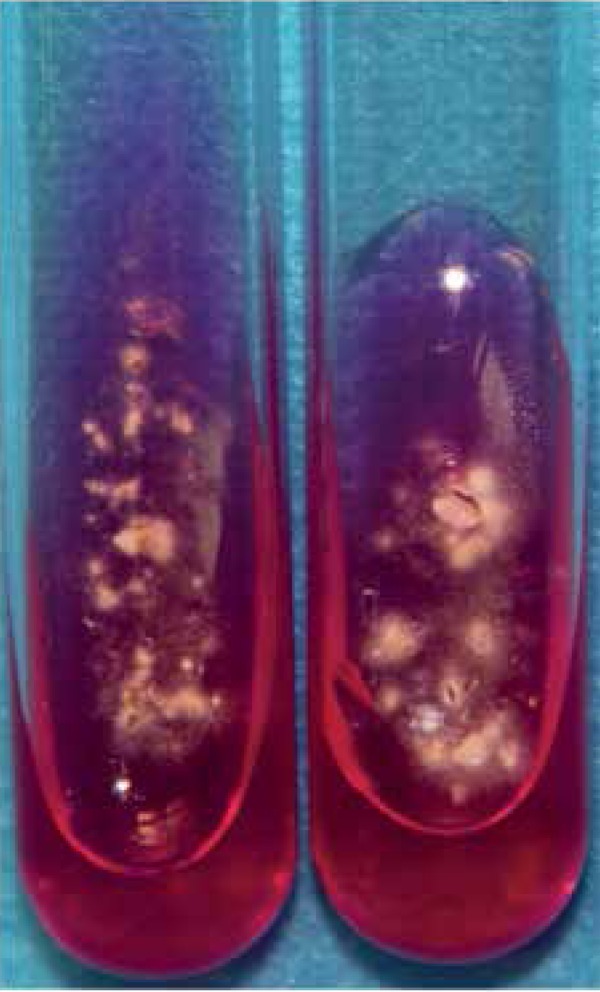
Urease activity test (rubescence was observed on urea agar slant).

Direct microscopic examination revealed hyaline hyphae. The culture on Sabouraud dextrose agar (SDA) at 28ºC revealed beige, powdery surfaces with orange-yellow pigment (Figure 2A). Microscopic examination with slide culture and scanning electron microscope observations of the colony indicated the morphology of *Trichophyton mentagrophytes* (Figure 2B, C). Urease activity on a urea agar slant (OxoidLtd, Hamp-shire, UK) incubated at 28ºC was assessed every day for 7 days (Figure 3). The identifications of molecular biology, as described by Kang et al., were confirmed by sequencing the nuclear ribosomal ITS region (GenBank accession number KF438222) and β-tubulin gene (GenBank accession number KU364381); they were consistent with *T. interdigitale*.^4,5^ The enzymatic activities of the isolated strain were determined using the semi-quantitative Api-Zym system (BioMerieux, Inc., Durham, NC, USA) as described by Yin et al. (Table 1).^6^ The girl was completely cured by oral treatment with terbinafine tablets (Lamisil, Beijing Novartis Pharmaceutical Ltd.), 250mg per day, combined with daily topical use of 1% naftifine-0.25% ketaconazole cream (Chongqing Huapont Pharmaceutical Co., Ltd.), after washing with 2% ketaconazole shampoo (Triatop, Xian-Janssen Pharmaceutical Ltd.) on the lesion (Figure 1C). No recurrence has been observed to date.

**Table 1:** The results of assayed enzymatic activities

NO.	Enzyme assayed	Results
1	Control	-
2	Alkaline phosphatase	+++++
3	Esterase(C4)	++
4	Esterase lipase(C8)	++
5	lipase(C14)	-
6	Leucine arylamidase	+++++
7	Valine arylamidase	++
8	Cystine arylamidase	-
9	Trypsin	-
10	α-chymotrypsin	-
11	Acid phosphatase	+++++
12	Naphtol-AS-BI-phosphohydrolase	+++++
13	α-galactosidase	-
14	β-galactosidase	-
15	β-glucuronidase	-
16	α-glucosaccharase	+++
17	β-glucosaccharase	+++++
18	N-acetyl-glucosaminidase	++++
19	α-mannosidase	+++++
20	β-fucosidase	-

## DISCUSSION

*T. interdigitale* is an anamorph of *A. vanbreuseghemii*, which belongs to the *T. mentagrophytes* complex. In tinea faciei, the fungus is the most frequently isolated dermatophyte, usually linked to contact with rabbits.^7^ In China, *T. interdigitale* infections have increased significantly and are associated with animals.^8^ The strain isolated from our case was macroscopically characterized by a beige, powdery surface and presented microscopically with numerous, thin-walled clavate macroconidia and round microconidia. These morphological features, combined with the strong inflammatory legion, indicate the zoophilic characteristics of the isolate in this case, which may originate from the rabbit.^9^ The Api Zym system revealed that *T. interdigitale* was capable of producing multiple, extracellular enzymes, similar to those previously reported.^10^ These main secreted enzymes may be associated with damage to host tissue such as hair and skin.^1^ In addition, tinea faciei is prone to presenting with atypical features, probably due to the complex anatomy of the face.^11^ Application of desonide and mometasone furoate usually alters its clinical appearance, leading to further misdiagnosis of tinea faciei. Consequently, the authors emphasize the importance of considering tinea in the differential diagnosis of all facial eruptions, especially with a plausible history of animal contact.^4^ Potassium hydroxide examination is a rapid, simple and necessary step in all cases of scaly facial lesions. Mycological culture not only further confirms the diagnosis, but also provides credible evidence to correct assertions when the result of KOH microscopy is negative.

In conclusion, tinea faciei usually presents a wide variety of symptoms especially after the application of corticosteroid and it can easily be misdiagnosed. Thus, the authors recommend that fungal infections should always be suspected in scaly eruptions on the face.
